# 5, 8, 11, 14-eicosatetraynoic acid suppresses CCL2/MCP-1 expression in IFN-γ-stimulated astrocytes by increasing MAPK phosphatase-1 mRNA stability

**DOI:** 10.1186/1742-2094-9-34

**Published:** 2012-02-18

**Authors:** Jee Hoon Lee, Hyunmi Kim, Joo Hong Woo, Eun-hye Joe, Ilo Jou

**Affiliations:** 1 Chronic Inflammatory Disease Research Center, Ajou University School of Medicine, Suwon 442-721, Korea; 2 Department of Pharmacology, Ajou University School of Medicine, Suwon 442-721, Korea

**Keywords:** CCL2/MCP-1, ETYA, MKP-1, HuR, IFN-γ, Astrocyte, Microglia

## Abstract

**Background:**

The peroxisome proliferator-activated receptor (PPAR)-α activator, 5,8,11,14-eicosatetraynoic acid (ETYA), is an arachidonic acid analog. It is reported to inhibit up-regulation of pro-inflammatory genes; however, its underlying mechanism of action is largely unknown. In the present study, we focused on the inhibitory action of ETYA on the expression of the chemokine, CCL2/MCP-1, which plays a key role in the initiation and progression of inflammation.

**Methods:**

To determine the effect of ETYA, primary cultured rat astrocytes and microglia were stimulated with IFN-γ in the presence of ETYA and then, expression of CCL2/MCP-1 and MAPK phosphatase (MKP-1) were determined using RT-PCR and ELISA. MKP-1 mRNA stability was evaluated by treating actinomycin D. The effect of MKP-1 and human antigen R (HuR) was analyzed by using specific siRNA transfection system. The localization of HuR was analyzed by immunocytochemistry and subcellular fractionation experiment.

**Results:**

We found that ETYA suppressed CCL2/MCP-1 transcription and secretion of CCL2/MCP-1 protein through up-regulation of MKP-1mRNA levels, resulting in suppression of c-Jun N-terminal kinase (JNK) phosphorylation and activator protein 1 (AP1) activity in IFN-γ-stimulated brain glial cells. Moreover, these effects of ETYA were independent of PPAR-α. Experiments using actinomycin D revealed that the ETYA-induced increase in MKP-1 mRNA levels reflected an increase in transcript stability. Knockdown experiments using small interfering RNA demonstrated that this increase in MKP-1 mRNA stability depended on HuR, an RNA-binding protein known to promote enhanced mRNA stability. Furthermore, ETYA-induced, HuR-mediated mRNA stabilization resulted from HuR-MKP-1 nucleocytoplasmic translocation, which served to protect MKP-1 mRNA from the mRNA degradation machinery.

**Conclusion:**

ETYA induces MKP-1 through HuR at the post-transcriptional level in a receptor-independent manner. The mechanism revealed here suggests eicosanoids as potential therapeutic modulators of inflammation that act through a novel target.

## Introduction

Inflammatory responses in the brain contribute to the pathogenesis of neurodegenerative disease, such as Alzheimer's disease, multiple sclerosis, and brain ischemia [1-4]. These responses are characterized by a sequential process involving the release of pro-inflammatory cytokines, increased expression of endothelial adhesion molecules and chemotactic factors, and activation of brain immune effector cells [5,6].

Microglia and astrocytes are representative immune cells in the brain. When microglia and astrocytes become activated by a variety of stimuli, they produce inflammatory cytokines and chemokines, which accelerate disease progression [7-11]. Among the inflammatory chemokines elaborated, glial cell-derived CCL2/MCP-1 is crucial: by promoting the migration and recruitment of inflammatory cells, it is primarily responsible for the initiation and progression of inflammatory responses [12]. In an animal model of prion disease, mice deficient in CCL2/MCP-1 showed a delayed onset of inflammatory disease and an increase in survival time [13]. CCL2/MCP-1 overexpression is also associated with a variety of disease states, including atherosclerosis [14,15] and ischemic stroke [16]. Therefore, intervention aimed at suppressing CCL2/MCP-1 expression is an emerging therapeutic strategy for the treatment of neurodegenerative diseases.

As a part of this therapeutic approach, peroxisome proliferator-activated receptors (PPARs) have received recent research attention. PPARs, which comprise three members--α, γ and β/δ--are ligand-activated transcription factors that form a subfamily of the nuclear receptor gene family. PPARs were originally reported to be highly expressed in adipocytes and were shown to play important roles in adipocyte differentiation, lipid biosynthesis, and glucose homeostasis [17,18]. However, subsequent studies have suggested that each PPAR subtype has anti-inflammatory effects in various cell types [19,20]. One of them, PPAR-α, exerts its anti-inflammatory functions by negatively regulating the expression of pro-inflammatory molecules [21-23]. In the brain, PPAR-α activators have been shown to inhibit the production of nitric oxide and the secretion of pro-inflammatory cytokines, including TNF-α, IL-1β, and IL-6 in glial cells [24-26]. Thus, PPAR-α activators have shown promising beneficial effects in several animal models of CNS disorders in which an inflammatory component is strongly implicated, such as multiple sclerosis, Parkinson's disease, Alzheimer's disease, and ischemic brain injury [27-29].

Although significant advances have been made in our understanding of the molecular mechanisms of PPAR-α activators during metabolic processes, much less is known about the anti-inflammatory mechanisms of PPAR-α activators during inflammation. For example, accumulating evidence suggests that PPAR activators act in a receptor-independent manner in various cell types. In one such case, the PPAR-γ activator, 15-deoxy-Δ12,14-prostaglandin J2 (15d-PGJ_2_), was shown to suppress inflammatory cytokines in a receptor-independent manner [30-32]. Activators of PPAR-α have also proven effective against experimental autoimmune encephalomyelitis (EAE) independent of PPAR-α [33]. Thus, PPAR activators are multifaceted modulators of inflammatory responses, functioning through both receptor-dependent and -independent mechanisms.

Here, we evaluated the anti-inflammatory effects of three fibrates, WY14643, fenofibrate, clofibrate, and the eicosanoid, 5,8,11,14-eicosatetraynoic acid (ETYA), as PPAR-α activators. Our results showed that ETYA, but not fibrates, acted through a PPAR-α-independent mechanism to suppress JNK-mediated CCL2/MCP-1 expression by inducing MKP-1 (MAPK phosphatase), a negative regulator of MAPK. ETYA-induced MKP-1 expression resulted from a HuR-mediated increase in MKP-1 mRNA stability. These findings suggest an additional therapeutic use of known anti-inflammatory agents based on a novel mechanism targeting post-transcriptional regulation.

## Materials and methods

### Reagents

IFN-γ was purchased from Calbiochem (Butler Pike, PA). Antibodies against phospho-JNK, phospho-MKP-1 and histone deacetylase (HDAC)1 were purchased from Cell Signaling (Beverly, MA). Anti-MKP-1, anti-HuR, anti-decaping protein (DCP)1α, anti-T cell internal antigen (TIA)1, anti-TIA1 related (TIAR), Lamin B, GAPDH and methyl CpG binding domain (MBD)3 antibodies were purchased from Santa Cruz Biotechnology (Santa Cruz, CA). ETYA, WY-14643, clofibrate, and 2-arachidonylglycerol (2-AG) were purchased from BIOMOL (Plymouth Meeting, PA). Fenofibrate and actinomycin D (Act D) were purchased from Sigma (St. Louis, MO).

### Cell culture

Primary microglia and astrocytes were cultured from the cerebral cortices of 1-day-old Sprague-Dawley rats. Cortices were triturated into single cells in minimal essential media (MEM) containing 10% fetal bovine serum (Hyclone, Logan, UT), plated onto 75-cm^2^T-flasks, and cultured for 2 weeks. Following the removal of microglia, primary astrocytes were isolated by trypsinization. Microglia and meningeal cells were depleted by incubating astrocytes in serum-free MEM for 2 days before use. Final cultures were shown to consist of more than 95% authentic astrocytes by glial fibrillary acidic protein (GFAP) staining.

### Reverse transcription-polymerase chain reaction (RT-PCR) and quantitative RT-PCR analysis

Total RNA was isolated using TRIzol (Invitrogen, Carlsbad, CA), and cDNA was prepared using Avian Myeloblastosis Virus reverse transcriptase (GenDEPOT, Barker, TX), according to the manufacturers' instructions. Conventional PCR was performed using 32 cycles of sequential reactions. The following primer pairs for the indicated targets were purchased from Bioneer (Daejeon, Korea): GAPDH, 5'-TCC CTC AAG ATT GTC AGC AA-3' (forward) and 5'-AGA TCC ACA ACG GAT ACA TT-3' (reverse); TNF-α, 5'-GTA GCC CAC GTC GTA GCA AA-3' (forward) and 5'-CCC TTC TCC AGC TGG GAG AC-3' (reverse); CCL2/MCP-1, 5'-ATG CAG GTC TCT GTC ACG CT-3' (forward) and 5'-CTA GTT CTC TGT CAT ACT GG-3' (reverse); MKP-1, 5'-AGG ACA CCA CAA GGC AGA C-3' (forward) and 5'-TGA TGG GGC TTT GAA GGT AG-3' (reverse); PPAR-α, 5'-TTC GGA AAC TGC AGA CCT-3' (forward) and 5'-TTA GGA ACT CTC GGG TGA T-3' (reverse); cannabinoid receptor (CB)1, 5'-TCC CAG GGA GAG GAG AGT GT-3' (forward) and 5'-GCC GTC ACC AGG TTT TCA CT-3' (reverse). For quantitative PCR, the amplification reactions were performed with KAPA SYBR qPCR master mix (KAPA Biosystems, Woburn, MA) according to the manufacturer's specifications. Amplification conditions were as follows: 40 cycles of 3 s at 95°C, 15 s at 55°C, and 15 s at 72 C. After amplification was complete, a melting curve was generated by heating at 1°C per second to 95°C. Melting curves were generated and data were quantitatively analyzed using Rotor Gene Q, version 1.7. The sequences of primers for quantitative RT-PCR were as follows: CCL2/MCP-1, 5'-ATG CAG TTA ATG CCC CAC TC-3' (forward) and 5'-TTC CTT ATT GGG GTC AGC AC-3' (reverse); MKP-1, 5'-TAG ACT CCA TCA AGG ATG CTG G-3' (forward) and 5'-GCA GCT CGG AGA GGT TGT GAT-3' (reverse); GAPDH, 5'-GGC CAA AAG GGT CAT CAT C-3' (forward) and 5'-GTG ATG CCA TGG ACT GTG G-3' (reverse).

### Western blot analysis

Cell lysates for Western blot analysis, prepared as previously described [32], were separated by SDS-PAGE and transferred to nitrocellulose membranes. Membranes were incubated with primary antibodies and HRP-conjugated secondary antibodies, and bands were visualized using an enhanced chemiluminescence system (Ab Frontier, Korea).

### EMSA

EMSAs were conducted following a previously reported method [32]. The oligonucleotide probe, 5'-CCT GAC TCC ACC TCT GGC-3', specific for the AP1 binding site of the rat CCL2/MCP-1 promoter (positions -129 to -111) was purchased from Bioneer. For supershift experiments, protein extracts were incubated with 0.2 μg of anti-c-Jun antibody (Santa Cruz Biotechnology) for 1 h prior to the addition of γ-^32^P-labeled probe.

### ELISA

Primary microglia and astrocytes were seeded onto 6-well plates. After incubating cells with IFN-γ in the presence or absence of ETYA or fibrates, 500 μl of cell-conditioned media was collected and assayed using rat CCL2/MCP-1 ELISA kits (BD Biosciences, San Diego, CA), according to the manufacturer's instructions.

### Construction of luciferase reporter plasmids and luciferase assay

Rat CCL2/MCP-1 promoter fragments corresponding to positions -3554 to +76 (pGL3-CCL2/MCP-1), -2355 to -81 (pGL3-κB-AP1-SP1), -2404 to -2062 (pGL3-κB) and -228 to +45 (pGL3-AP1-Sp-1) were amplified by PCR using rat genomic DNA as a template and specific primer sets. Transient transfections were performed using Lipofectamine 2000 reagents (Invitrogen) as described by the manufacturer. After incubating transfected cells for 48 h, luciferase activity was measured using a luminometer (PerkinElmer Vitor^3^) and normalized to β-galactosidase activity (measured at *A*_420_)

### ChIP assay

ChIP assays were performed as described previously [32]. Cell supernatants were immunoprecipitated with anti-c-Jun antibodies overnight at 4°C; protein-bound, immunoprecipitated DNA was recovered by phenol/chloroform extraction and then amplified by PCR using the primer pair, 5'-TTC CAC TCT CCA TCG CTC AT- 3' (forward) and 5'-TCT GCA TTT CTA GCG GCT CT-3' (reverse), spanning the promoter region of CCL2/MCP-1 (position -155 to -71) containing AP1 elements.

### Immunostaining and confocal microscopy

Astrocytes cultured on poly-D-lysine-coated coverslips were fixed with methanol at -20°C for 30 min. Fixed cells were incubated with anti-HuR and anti-GFAP (Sigma) antibodies at 4°C overnight, and then with fluorescein- or rhodamine-conjugated secondary antibodies (Molecular Probes, Eugene, OR) for 2 h. Coverslips were slide-mounted and observed under a confocal microscope (Zeiss, Germany).

### Synthesis and transfection of small interfering RNA

siRNA duplex oligonucleotides targeting MKP-1 (5'-CCA ATT GTC CTA ACC ACT T-3'), CB1 (5'-CGA AGG UGA CCA UGU CUG UTT-3'), HuR (5'-GAU GCC AAC UUG UAC AUC ATT-3') and PPAR-α (5'-GGC UAA AGC UGG CGU ACG AUU-3') were chemically synthesized by Bioneer (Korea). Confluent astrocytes and microglia were transfected with siRNA oligonucleotides using Lipofectamine RNAiMAX (Invitrogen), according to the manufacturer's instructions. All assays were performed at least 48 h after siRNA transfection.

### Phosphatase assay

Cell extracts were prepared by immunoprecipitation, as previously described [32]. Briefly, lysates (300 μg) were incubated with an anti-MKP-1 antibody (1 μg) at 4°C overnight, and precipitated by incubating with protein G-agarose beads (Upstate Biotechnology) for 2 h at 4°C. Phosphatase activity was measured in two ways. In the first, MKP-1 activity was measured by incubating immunoprecipitated proteins with the substrate, *p*-nitrophenylphosphate (*p*-NPP) (Sigma), for 4 h at 37°C followed by spectrophotometric analysis at 405 nm. In the second, specific p-JNK-linked MKP-1 activity was measured by incubating immunoprecipitated proteins with lysates from IFN-γ-stimulated astrocytes (as substrates). Bead-protein conjugates and lysates were then boiled, and the resulting eluates were analyzed by Western blotting using antibodies against MKP-1 or phospho-JNK.

### Statistical analysis

Differences among groups were determined using one-way ANOVA. A *p-*value of 0.05 was considered statistically significant. Values are presented as means ± SEMs or ± SDs

## Results

### ETYA suppresses CCL2/MCP-1 transcription and protein secretion by inhibiting AP1 signaling in IFN-γ-activated brain astrocytes

**Figure 1:**
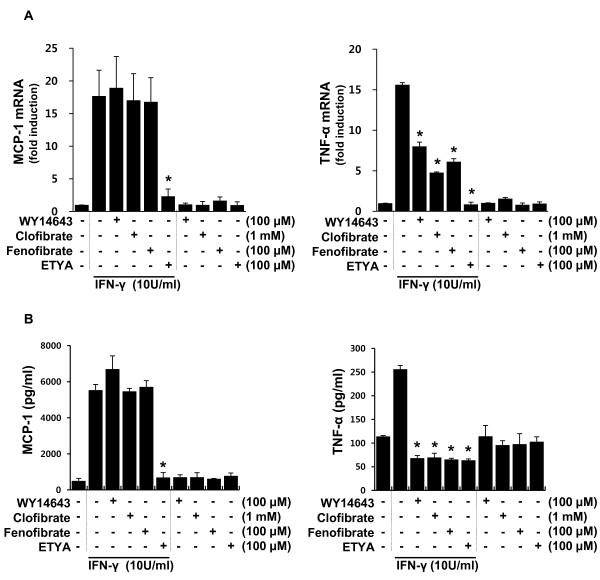
**ETYA, but not fibrates, reduces CCL2/MCP-1 transcript levels and protein release in IFN-γ-stimulated astrocytes**. **(A and B) **Primary astrocytes were stimulated with IFN-γ for 3 h (**A**) or 12 h (**B**) in the presence of the indicated levels of individual PPAR-α activators. Then, CCL2/MCP-1 and TNF-α mRNA levels and protein secretion into media were determined using qRT-PCR and ELISA, respectively. Data are presented as means ± SDs or SEMs of three independent experiments (*p < 0.01 versus IFN-γ group).

First, we screened the anti-inflammatory profiles of PPAR-α activators in IFN**-γ-**stimulated brain astrocytes using RT-PCR analyses and ELISAs. Astrocytes were stimulated with IFN-γ (10 U/ml) in the absence or presence of one of four PPAR-α activators: the three fibrates, WY14643, clofibrate and fenofibrate; and the eicosanoid, ETYA. Effective concentration of individual agent was determined according to a dose test result (Additional file 1: Figure S1). TNF-α and CCL2/MCP-1 transcript levels and protein released into media were measured 3 and 12 h after IFN-γ treatment, respectively. TNF-α transcript and released protein levels were suppressed by all PPAR-α activators, whereas those of CCL2/MCP-1 were inhibited by ETYA, but not by fibrates (Figure 1A and 1B).

**Figure 2:**
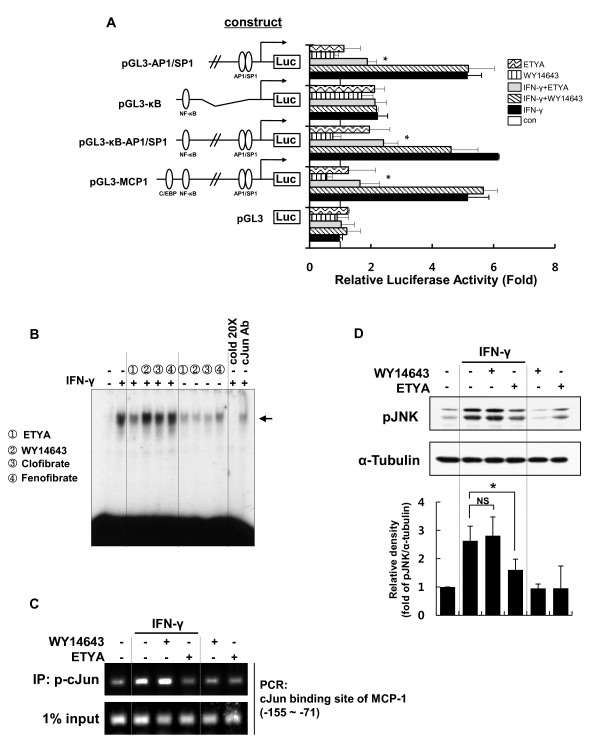
**ETYA-induced CCL2/MCP-1 suppression depends on the inhibition of JNK-AP1 signals**. **(A) **Astrocytes were transiently transfected with the indicated 5'-deleted CCL2/MCP-1 promoter constructs and incubated for 48 h. After stimulation with IFN-γ in the presence of the indicated activators for 6 h, cells were harvested and luciferase activity was measured and plotted as fold-induction over untreated controls. Data are presented as means ± SEMs for three independent experiments (*p < 0.05 versus IFN-γ group). **(B) **Astrocytes were stimulated with IFN-γ in the presence of the indicated PPAR-α activators, and nuclear factor binding activities of AP1 were measured by EMSA using oligonucleotide probes specific for the AP1 site of the rat CCL2/MCP-1 promoter (-129 to -111). The arrows represent specific DNA-protein complexes. **(C) **Astrocytes were treated with ETYA or WY-14643 and prepared for ChIP assays (see Materials and Methods for details). "Input" indicates control PCR and shows the amount of CCL2/MCP-1 promoter DNA present in each sample before ChIP. **(D) **After stimulating astrocytes with IFN-γ for 2 h in the presence of ETYA or WY-14643, JNK activity was measured by Western blot analysis using an anti-phospho-JNK antibody. The bar graph represents the intensities of pJNK bands normalized against those of α-tubulin (bottom panel). The data are presented as means ± SDs of three independent experiments. *p < 0.05, NS; non significant.

Because we have previously shown that CCL2/MCP-1 expression is critically regulated by JNK/AP1 signaling in brain astrocytes [32], we examined whether ETYA acted through inhibition of JNK/AP1 to suppress CCL2/MCP-1 expression. Using variably deleted rat CCL2/MCP-1 promoter/luciferase reporter gene constructs (refer to Materials and Methods), we tested whether fibrates and ETYA affected CCL2/MCP-1 transcription. IFN-γ-induced increases in the luciferase activity of promoters containing AP1/SP1 sites were suppressed by ETYA, but not by WY14643 (Figure 2A). To further evaluate the effect of ETYA, we performed EMSAs and ChIP assays. These assays confirmed that ETYA, but not fibrates, effectively inhibited c-Jun binding to the promoter of the CCL2/MCP-1 gene (Figure 2B and 2C). AP1 is composed of JNK-phosphorylated c-Jun homodimers or heterodimers with c-Fos [34]. Thus, we examined whether ETYA acted at the level of JNK phosphorylation. JNK phosphorylation, which was evident within 2 h of IFN-γ stimulation, was markedly suppressed by ETYA, but not by WY14643 (Figure 2D). To confirm the functional relevance of pJNK suppression by ETYA, we checked whether ETYA affected the localization of MBD3 and HDAC1, which were known to repress target gene expression by binding to AP-1 site of target gene in a JNK phosphorylation-dependent manner [35]. Using ChIP assay, we observed that binding of MBD3 and HDAC1 to the MCP-1 promoter is increased in ETYA-treated group as compared to IFN-γ-treated group. These results confirm that ETYA-mediated JNK inactivation functionally affect MCP-1 gene expression (Additional file 2: Figure S2). Collectively, these results indicate that ETYA, but not fibrates, effectively suppressed CCL2/MCP-1 expression in IFN-γ-stimulated astrocytes by inhibiting AP1 signaling.

### The suppressive actions of ETYA on JNK/AP1 are mediated by MKP-1 and are independent of PPAR-α

**Figure 3:**
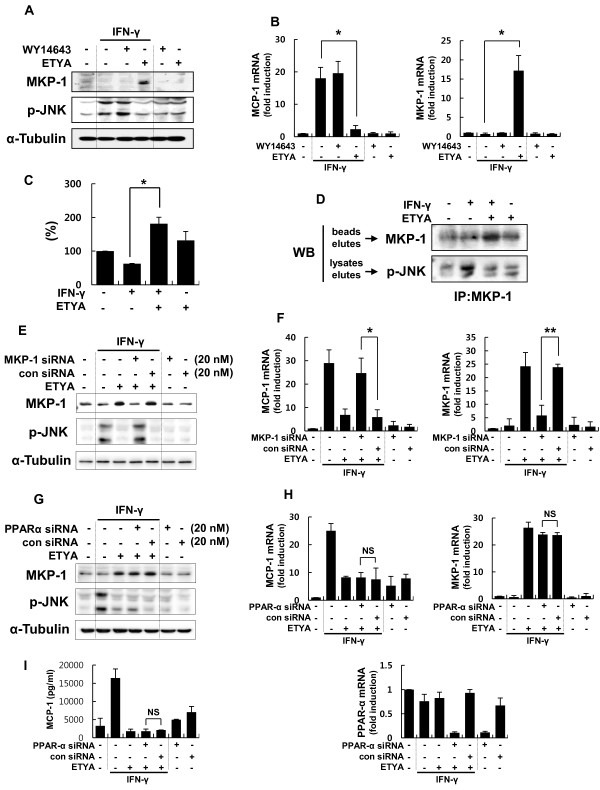
**ETYA acts in a PPAR-α-independent manner to suppress CCL2/MCP-1 expression through induction of MKP-1 expression and phosphatase activity**. **(A and B) **ETYA- or WY-14643-treated astrocytes were stimulated with IFN-γ for 2 h. MKP-1 protein levels and JNK phosphorylation were analyzed by Western blotting (**A**), and MKP-1 and CCL2/MCP-1 transcript levels were determined by qRT-PCR (**B**). **(C and D) **Cell lysates immunoprecipitated with an anti-MKP-1 antibody were incubated with *p*-NPP for 4 h and then analyzed spectrophotometrically at 405 nm (**C**), or incubated with lysates from IFN-γ-activated astrocytes followed by Western blot analysis of eluates (**D**). **(E-I) **Primary astrocytes were transfected with an MKP-1-specific (**E **and **F**) or PPAR-α-specific siRNA duplex (G-I) or a nonsilencing control siRNA. Forty-eight h after transfection, cells were stimulated with IFN-γ for 2 h or 12 h in the absence or presence of ETYA. MKP-1 protein levels and phospho-JNK, and transcript levels of MKP-1 and CCL2/MCP-1 were determined by Western blot analysis (**E **and **G**) and qRT-PCR (**F **and **H**). MCP-1 protein secretion was analyzed by ELISA (**I**). Efficiency of siRNA-mediated PPAR-α silencing was demonstrated by monitoring expression of acetyl CoA synthase (ACS), a PPAR-α-dependent gene (data not shown). Values are means ± SDs. of three independent experiments. *p < 0.05, ** *p *< 0.01, NS; non significant.

We have previously reported that MKP-1, which is a negative regulator of JNK, is critically involved in CCL2/MCP-1 expression [32]. On the basis of these observations, we examined whether the regulation of JNK phosphorylation and CCL2/MCP-1 expression in IFN-γ-stimulated astrocytes by ETYA was a consequence of ETYA-induced modulation of MKP-1. qRT-PCR and Western blot analyses showed that MKP-1 mRNA and protein, respectively, were induced within 2 h in the presence of ETYA. The increased level of MKP-1 was significantly correlated with a decrease in JNK phosphorylation and suppression of CCL2/MCP-1 expression (Figure 3A and 3B). Additionally, MKP-1 phosphatase activity was markedly increased with ETYA treatment (Figure 3C). These results were confirmed in a more specific phosphatase assay using lysates containing excess phospho-JNK as a MKP-1 substrate instead of the synthetic substrate, *p-*NPP (see Materials and Methods for details) (Figure 3D). Moreover, these effects of ETYA on CCL2/MCP-1 expression were reversed by siRNA-mediated MKP-1 knockdown, indicating that MKP-1 likely mediates these effects (Figure 3E and 3F). Taken together, these results clearly indicate that ETYA induces an increase in the expression level and enzymatic activity of MKP-1.

Because ETYA is known as a PPAR-α activator [36], we examined whether the actions of ETYA on MKP-1 are receptor-dependent or independent. As shown in Figure 3G and 3H, ETYA-induced MKP-1 expression was not reversed by siRNA-mediated PPAR-α knockdown; CCL2/MCP-1 expression levels were also unaffected (Figure 3I) suggesting that ETYA acted in a receptor-independent manner.

### CCL2/MCP-1 suppression by ETYA in brain microglia is also mediated by the induction of MKP-1

**Figure 4:**
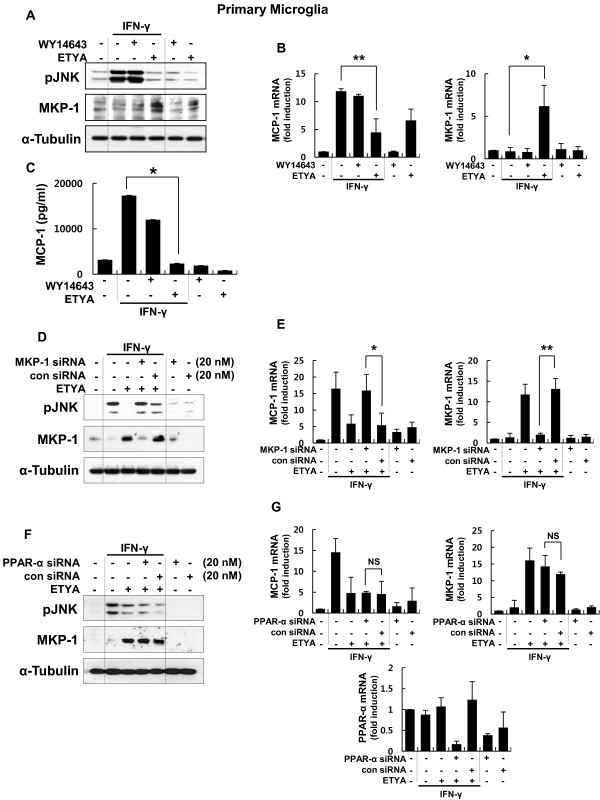
**ETYA suppresses CCL2/MCP-1 expression by inducing MKP-1 expression in brain microglia**. **(A **- **C) **Primary microglia were stimulated with 10 U/ml IFN-γ for 2 h or 12 h in the presence of ETYA or WY-14643. MKP-1 protein expression and JNK phosphorylation were analyzed by Western blotting (**A**), and MKP-1 and CCL2/MCP-1 transcript levels and protein release were examined by qRT-PCR (**B**) and ELISA (**C**). **(D **- **G) **Rat microglia were transfected with a siRNA duplex specific for MKP-1 (**D **and **E**) or PPAR-α (**F **and **G**). After 48 h, cells were stimulated with IFN-γ for 2 h in the presence or absence of ETYA. MKP-1 protein and JNK phosphorylation were analyzed by Western blotting (**D **and **F**), and MKP-1 and CCL2/MCP-1 transcript levels were examined by RT-PCR (**E **and **G**). Values are means ± SDs of three independent experiments. *p < 0.05, **p < 0.01, NS; non significant.

Next, we examined whether the differential effects of fibrates and ETYA on CCL2/MCP-1 and MKP-1 expression were evident in rat microglia, which are resident immune effector cells in the CNS. Rat microglia were stimulated with IFN-γ in the absence or presence of WY14643 or ETYA. Consistent with the results obtained from astrocytes, ETYA inhibited IFN-γ-induced increases in the levels of CCL2/MCP-1 transcripts and protein, and simultaneously induced MKP-1 levels (Figure 4A-C). These effects were reversed by MKP-1 knockdown (Figure 4D and 4E), but not by siRNA-mediated PPAR-α knockdown (Figure 4F and 4G). In contrast to the data obtained with glial cells, fibrates and ETYA induced equivalent suppression of CCL2/MCP-1 expression in rat peritoneal macrophages (Additional file 3: Figure S3), suggesting that the differential effects of fibrates and ETYA are glial-cell specific.

### ETYA increases MKP-1 mRNA stability by inducing HuR cytoplasmic translocation

**Figure 5:**
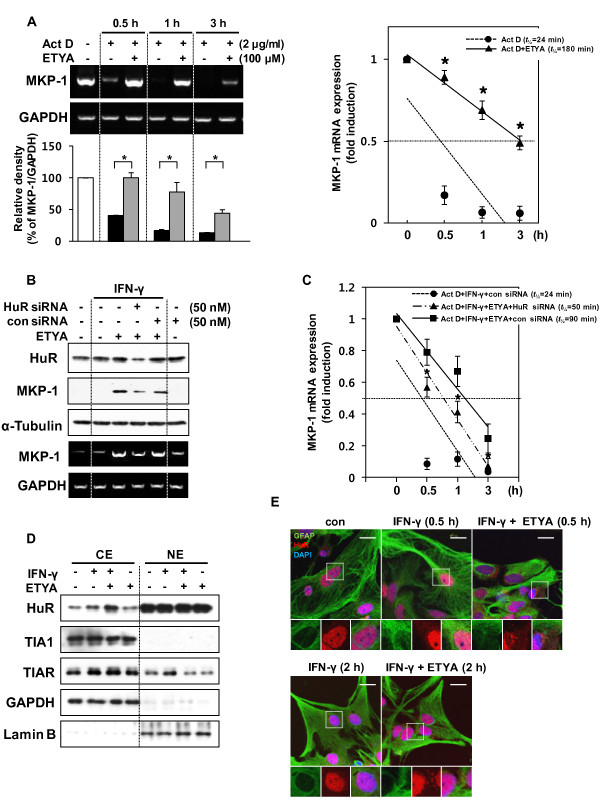
**The ETYA-induced increase in MKP-1 expression involves a HuR-mediated increase in MKP-1 mRNA stability resulting from HuR cytoplasmic translocation**. **(A) **Astrocytes were treated with Act D for the indicated periods in the presence of ETYA, after which MKP-1 mRNA levels were determined by conventional RT-PCR (left, upper panel) and qRT-PCR (right panel). The bar graph represents the intensities of MKP-1 bands normalized against those of GAPDH (left, bottom panel). All data are presented as means ± SDs of three independent experiments (*p < 0.05). **(B and C) **Astrocytes were transfected with a HuR-specific or nonsilencing control siRNA. Forty-eight h after transfection, cells were stimulated with IFN-γ in the absence or presence of ETYA. MKP-1 mRNA and protein levels were determined by RT-PCR and Western blot analysis (B), and MKP-1 mRNA stability was determined by qRT-PCR (**C**). The data are presented as means ± SDs of three independent experiments (*p < 0.05 versus IFN-γ + ETYA + con siRNA group). **(D) **Astrocytes were stimulated with IFN-γ in the presence or absence of ETYA, and nuclear extracts (NE) and cytosolic extracts (CE) were prepared for Western blotting. **(E) **Confocal microscopic images of astrocytes immunostained with antibodies against HuR, GFAP (astrocyte marker), and DAPI (nuclear marker), under the indicated conditions. Insets are magnified images of the corresponding boxed regions. Scale bars, 20 μm.

MKP-1 expression is controlled at multiple levels. Several transcription factors, including SP1, SP3 and AP1, are known to influence MKP-1 expression at the transcriptional level in response to stress stimuli [37,38]. However, relatively little is known about the post-transcriptional regulation of MKP-1, despite the fact that MKP-1 is an early response gene and, thus, is likely to be encoded by a short-lived mRNA [39,40]. Because treatment with ETYA maintained elevated MKP-1 mRNA levels for up to 5 h (data not shown), we hypothesized that these agents increased MKP-1 mRNA levels through a post-transcriptional mechanism. To investigate this possibility, we treated astrocytes with the transcriptional inhibitor, actinomycin D (Act D), in the absence or presence of ETYA. MKP-1 mRNA levels were reduced in a time-dependent manner by Act D treatment. This effect was reversed by ETYA (Figure 5A), which had no effect on MKP-1 mRNA levels. These results suggest that ETYA regulates MKP-1 mRNA stability at the post-transcriptional level.

MKP-1 mRNA stabilization is mediated by various RNA binding proteins (RNA-BPs), including NF-90 and HuR [41]. Previous study showed that HuR influences MKP-1 stability through binding to MKP-1 AU-rich elements (AREs) in the 3'-untranslated region (UTR) [41-43]. We thus inferred that HuR was involved in ETYA-induced MKP-1 mRNA stabilization mechanism. To determine this possibility, we tested the effects of siRNA against HuR on MKP-1 mRNA levels. As shown in Figure 5B and 5C, siRNA-mediated HuR knockdown inhibited the stabilizing effect of ETYA on MKP-1 mRNA. Because the influence of HuR on the expression of target mRNAs is linked to its cytoplasmic export, we next examined whether ETYA induced HuR cytoplasmic translocation. The results of subcellular fractionation experiments showed that ETYA increased the cytoplasmic translocation of HuR, but had no effect on other RNA-BPs (Figure 5D); this effect was also confirmed using an immunocytochemical approach. Namely, HuR was shown to retain its nuclear localization in untreated astrocytes, whereas in cells treated with ETYA, HuR was partly displaced into the cytoplasm (0.5 h), and over time, appeared in a spotted fashion in the cytoplasm (2 h) (Figure 5E). Taken together, these results suggest that ETYA induces MKP-1 mRNA stability by increasing HuR cytoplasmic translocation.

## Discussion

**Figure 6:**
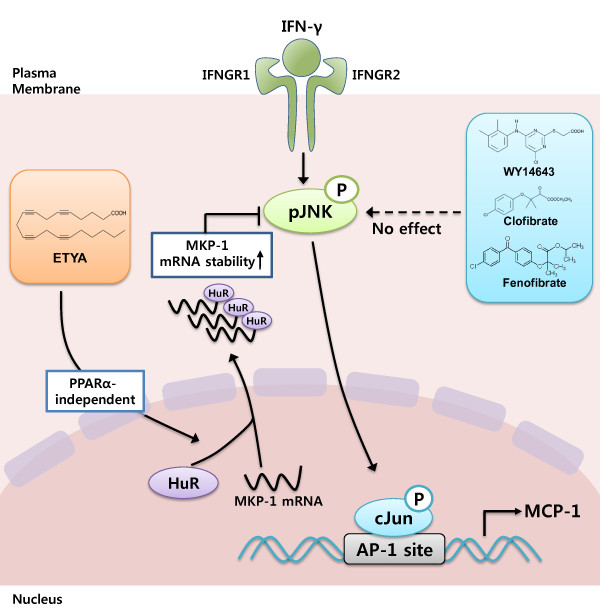
**A model showing the anti-inflammatory mechanism of ETYA in IFN-γ-stimulated glial cell**. IFN-γ induces JNK/Jun phosphorylation and increases CCL2/MCP-1 gene expression. ETYA, but not fibrates, increases MKP-1 mRNA stability by inducing HuR cytoplasmic shuttling, and thereby suppresses IFN-γ-induced JNK signaling and CCL2/MCP-1 expression.

Although anti-inflammatory effects of PPAR-α activators in various cell types have been reported, the underlying mechanisms are largely unknown. Different PPAR-α activators have distinctive effects on inflammation depending on cell type or stimulus. Accordingly, we questioned which of these various agents had specific anti-inflammatory effects in brain glial cell, and sought to identify the underlying mechanism. In this study, we demonstrated that ETYA, but not fibrates, act in a PPAR-α-independent manner to suppress the expression of CCL2/MCP-1 by increasing HuR-mediated-MKP-1 mRNA stability. This pathway is summarized in Figure 6.

MKP-1, a dual specificity (Ser/Thr or Thr/Tyr) protein phosphatase, is responsible for the inactivation of JNK/p38, and therefore controls MAPK-dependent inflammation during the innate immune response [44]. The roles of MKP-1 in inflammation are relatively well identified, and our previous studies also showed that 15d-PGJ_2_, a PPAR-γ activator, suppressed brain glial cell-mediated inflammatory responses through regulation of MKP-1 [32]. However, the detailed mechanism by which MKP-1 expression was regulated by PPAR activators remained to be established. MKP-1 expression could be regulated at transcriptional or post-transcriptional levels. Although several transcription factors, including SP1, SP3 and AP1, are known to be involved in the transcriptional regulation of MKP-1 in response to growth factors and stress stimuli [37,38], it is unlikely that ETYA acts through such a mechanism because it had no effect on the activity of these transcription factors (data not shown). Instead, because MKP-1 is an early response gene with a short-lived mRNA, it is likely to be regulated post-transcriptionally. Our study showed that ETYA treatment maintained MKP-1 mRNA levels for up to 5 h after mRNA synthesis was blocked with Act D, revealing that ETYA acted at the post-transcriptional level to increase MKP-1 mRNA stability (Figure 5).

The transient, stimulus-driven stabilization of early response transcripts is controlled by sequence-specific RNA-BPs that influence mRNA metabolism [45]. RNA-BPs that inhibit mRNA decay include the embryonic lethal abnormal vision (Hu/elav) family of RNA-BPs, consisting of the ubiquitous HuR (HuA) protein and the primarily neuronal proteins, HuB (Hel-N1), HuC, and HuD [46]. The most extensively studied member, HuR, binds to the AREs present in 3'-UTR of target mRNA and subsequently translocated to the cytoplasm, where it increases the half-life of many mRNAs, such as cyclooxygenase-2 (COX-2), inducible nitric oxide (iNOS), and MKP-1 [41,47,48]. We confirmed that the ETYA-induced increase in MKP-1 stability is mediated by HuR and occurs through the cytoplasmic translocation of HuR. Interestingly, we observed that this cytoplasmic HuR tended to form spot-like aggregates over time (Figure 5). Using a confocal imaging system, we confirmed that these aggregates were co-localized with stress granules (SGs), but not processing bodies (PBs) (Additional file 4: Figure S4). SGs are transient, dynamic cytoplasmic sites containing aggregates of mRNA. Unlike PBs, which contain mRNA decay machinery, SGs consist of translation initiation factors, small ribosomal subunits, and a diverse group of mRNAs and proteins, and are linked to RNA metabolism. Some RNA-BPs, such as tristetraprolin (TTP) and butyrate response factor (BRF)1, a subunit of the RNA polymerase III transcription initiation factor IIIB, destabilize specific transcripts, whereas other stabilizing proteins, such as HuR, bind transcripts for export or storage until stress conditions are alleviated [49]. Thus, SGs are thought to be passive repositories of the untranslated mRNAs that accumulate during stress, and serve to enable reinitiation of translation when environmental conditions improve. In this study, we speculated that ETYA induced a process in which cytosol-exported HuR caused accumulation of MKP-1 mRNA transcripts in SGs, preventing them from decaying under unfavorable conditions.

These observations give rise to a question: how does ETYA induce HuR export to the cytoplasm? Recent reports have shown that phosphorylation of HuR within the hinge region is mediated by protein kinase C (PKC; isoforms α and δ) and cyclin-dependent kinase (Cdk) and these are linked to the nucleocytoplasmic translocation of HuR [50-52]. Phosphorylation of HuR at S158 and S221 by PKC has been implicated in the translocation of HuR to the cytoplasm, whereas Cdk1-mediated phosphorylation at HuR at S202 helps to maintain HuR in the nucleus. Thus, the effects of ETYA on MKP-1 could be achieved by HuR phosphorylation via PKC or Cdk1. To verify this possibility, we first examined whether ETYA induces HuR serine phosphorylation. Immunoprecipitation experiments using nuclear/cytoplasmic extracts revealed that ETYA induced HuR serine phosphorylation (Additional file 5: Figure S5). Although it is reasonable to suppose that these modifications are linked to cytoplasmic translocation, we did not determine which serine residues of HuR are phosphorylated by ETYA. Thus, further studies are needed to clarify which serine sites are phosphorylated by ETYA and which upstream molecules (PKC or Cdk) are regulated by ETYA.

Recently, accumulating evidence suggests that cannabinoids act (directly or indirectly) to suppress inflammation-dependent neurodegeneration via inhibition of pro-inflammatory cytokine expression, and that of reactive oxygen intermediates; several cellular pathways are involved [53,54]. Of particular interest in this context are recent reports addressing functional interactions between cannabinoids and anti-inflammatory nuclear receptors, including PPARs [55-57]. These studies suggest that a novel mechanism potentiating anti-inflammatory capacity upon brain inflammation may be in play. As endocannabinoids such as anandamide (AEA) and 2-AG are derived from arachidonic acid synthesized within the body, it is likely that endocannabinoids and ETYA share structural and functional similarities [58]. Moreover, recent studies have shown that endocannabinoids not only inhibit the JNK activation induced by inflammatory stimuli [59,60], but also induce expression of MKP-1 [61,62]. We found that both 2-AG and ETYA suppressed IFN-γ-induced JNK phosphorylation and CCL2/MCP-1 expression, and inhibited MKP-1 synthesis in rat astrocytes. Further, we determined that the mechanism of 2-AG action was CB1 receptor-dependent but PPAR-α-independent, and that ETYA action did not require either receptor. This means that the signaling mechanisms regulating the level of MKP-1 expression differ in nature (Additional file 6: Figure S6). This results suggest another important point that the eicosanoid, ETYA, and the cannabinoid derivative, 2-AG, which evidence suggests share some structural and functional similarities [58], regulate MKP-1 in brain glial cells through different mechanisms. Thus, although both agents increased MKP-1 expression, the underlying mechanisms were quite different, with ETYA increasing MKP-1 mRNA levels post-transcriptionally, and 2-AG likely regulating MKP-1 expression at the transcriptional level (Figure S6G, and data not shown). Although the molecular targets of these agents upstream of MKP-1 are not yet known, it is important to note that these structurally similar compounds actively regulate MKP-1 levels through transcriptional and post-transcriptional mechanisms in a target- and cell-specific manner. In this context, identifying the specific underlying mechanism could provide a novel therapeutic target for anti-inflammatory drugs. Importantly, the development of such drugs that target different mechanisms could not only reduce side effects on other organs, but could also eliminate undesirable induction of receptor-dependent target genes.

## Conclusions

In this study, we demonstrated a novel anti-inflammatory mechanism of ETYA, a PPAR-α ligand, by increasing HuR-mediated-MKP-1 mRNA stability that leads to the specific suppression of CCL2/MCP-1 during inflammatory processes. ETYA induced HuR-MKP-1 nucleocytoplasmic translocation, which subsequently, served to protect MKP-1 mRNA from the mRNA degradation machinery. These findings establish a novel mechanism in which ETYA increases MKP-1 expression through HuR at the post-transcriptional level in a receptor-independent manner.

## Competing interests

The authors declare that they have no competing interests.

## Authors' contributions

JHL and IJ have contributed to the conception and experimental design of the study; JHL, HMK and JHW carried out experimental work; JHL, EJ and IJ analyzed the data; JHL and IJ wrote the manuscript. All authors have read and approved the final manuscript.

## Supplementary Material

Additional file 1 **Figure S1**. Dose-related effect of PPAR-α activators on inflammatory mediators expression in IFN-γ-stimulated astrocytes. Primary astrocytes were stimulated with IFN-γ for 2 h in the presence of the indicated concentrations of individual PPAR-α activators. Then, MCP-1 and TNF-α mRNA levels were determined using RT-PCR. Click here for file

Additional file 2 **Figure S2**. ETYA increases recruitment of Mbd3/HDAC1 to the AP-1 site of MCP-1 through JNK unpohosphoryation. Astrocytes were treated with ETYA or SP600125, a chemical inhibitor of JNK phosphorylation, and prepared for ChIP assays. "Input" indicates control PCR and shows the amount of CCL2/MCP-1 promoter DNA present in each sample before ChIP. Click here for file

Additional file 3 **Figure S3**. WY14643 and ETYA induce equivalent reductions in MCP-1 transcript levels and protein release in IFN-γ-stimulated peritoneal macrophages. (A and B) Peritoneal macrophages were stimulated with IFN-γ for 2 or 12 h in the presence of the indicated levels of individual PPAR-α activators. Then, MCP-1 and MKP-1 mRNA levels (A) and MCP-1 protein secretion into media (B) were determined using RT-PCR and ELISA, respectively. ELISA data are presented as means ± SEMs of three independent experiments (*p < 0.01 versus IFN-γ group). Click here for file

Additional file 4 **Figure S4**. ETYA promotes HuR translocation to stress granules (SGs), not processing bodies (PBs). (A and B) Confocal microscopic images of astrocytes immunostained with HuR, TIA1 (SG marker) (A), or DCP1α (PB marker) (B) under the indicated conditions. Insets are magnified images of the corresponding boxed regions. Scale bars, 20 μm. Arrows indicate co-localization of HuR and TIA1. Click here for file

Additional file 5 **Figure S5**. ETYA increases HuR serine phosphorylation. Astrocytes were stimulated with IFN-γ in the presence or absence of ETYA, and nuclear extracts (NE) and cytosolic extracts (CE) were immunoprecipitated with an anti-phospho-serine antibody. Phosphorylation of HuR in immunoprecipitates was measured by Western blot analysis using an anti-HuR antibody. Click here for file

Additional file 6 **Figure S6**. 2-AG also suppresses CCL2/MCP-1 expression by inducing MKP-1 expression. (A and B) Astrocytes were stimulated with IFN-γ for 2 h in the absence or presence of the indicated dose of 2-AG. MKP-1 protein expression and JNK phosphorylation were analyzed by Western blotting (A), and MKP-1 and CCL2/MCP-1 transcript levels were examined by qRT-PCR (B). (C-F) Cells were transfected with siRNA duplexes specific for PPAR-α (C and D) or CB1 (E and F), and 48 h later were stimulated with IFN-γ for 2 h in the presence of 2-AG or ETYA. MKP-1 protein level and JNK phosphorylation were analyzed by Western blotting (C and E), and MKP-1 and CCL2/MCP-1 transcript levels were examined by qRT-PCR (D and F). (G) Astrocytes were treated with Act D for the indicated periods in the presence of 2-AG, after which MKP-1 mRNA levels were determined by qRT-PCR. Click here for file
