# Supplementary figures and images for: 5, 8, 11, 14-eicosatetraynoic acid suppresses CCL2/MCP-1 expression in IFN-γ-stimulated astrocytes by increasing MAPK phosphatase-1 mRNA stability
Source: J Neuroinflammation. 2012 Feb 18;9:34. doi: 10.1186/1742-2094-9-34 (PMC3308915; doi:10.1186/1742-2094-9-34)

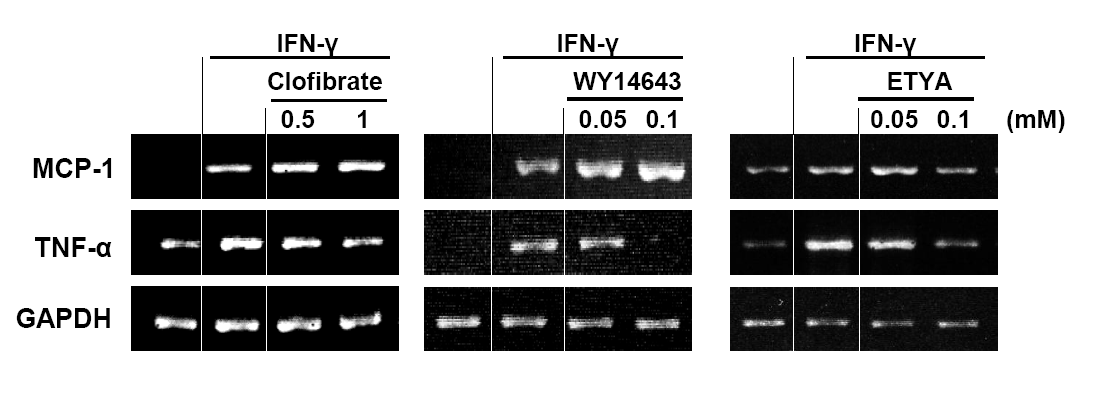

Supplement: Additional file 1 — Figure S1. Dose-related effect of PPAR-α activators on inflammatory mediators expression in IFN-γ-stimulated astrocytes. Primary astrocytes were stimulated with IFN-γ for 2 h in the presence of the indicated concentrations of individual PPAR-α activators. Then, MCP-1 and TNF-α mRNA levels were determined using RT-PCR. [file 1742-2094-9-34-S1.BMP]

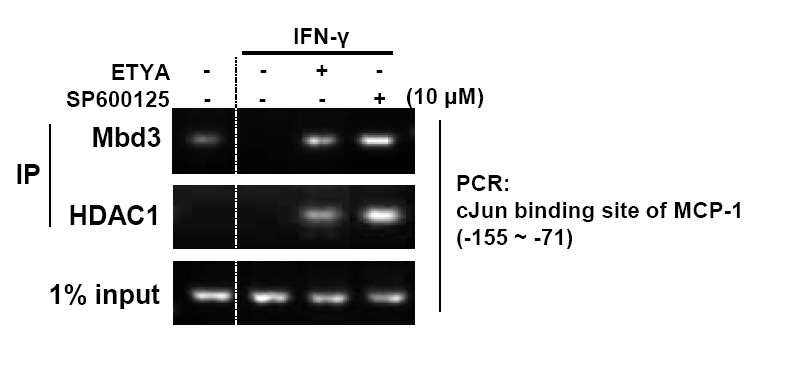

Supplement: Additional file 2 — Figure S2. ETYA increases recruitment of Mbd3/HDAC1 to the AP-1 site of MCP-1 through JNK unpohosphoryation. Astrocytes were treated with ETYA or SP600125, a chemical inhibitor of JNK phosphorylation, and prepared for ChIP assays. "Input" indicates control PCR and shows the amount of CCL2/MCP-1 promoter DNA present in each sample before ChIP. [file 1742-2094-9-34-S2.BMP]

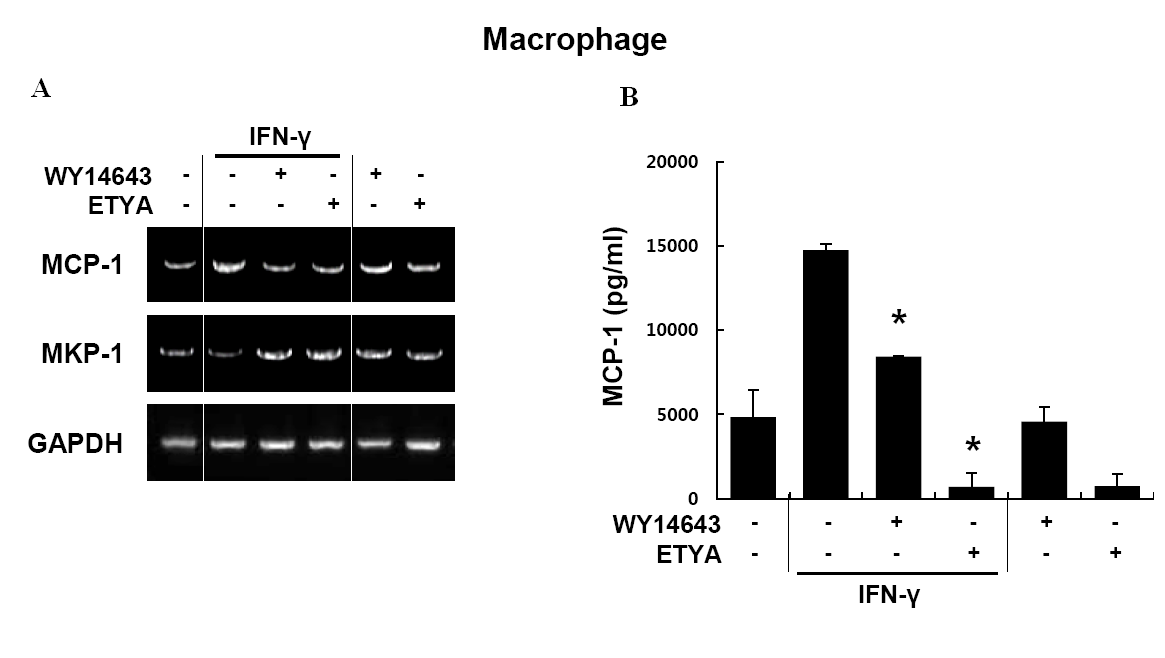

Supplement: Additional file 3 — Figure S3. WY14643 and ETYA induce equivalent reductions in MCP-1 transcript levels and protein release in IFN-γ-stimulated peritoneal macrophages. (A and B) Peritoneal macrophages were stimulated with IFN-γ for 2 or 12 h in the presence of the indicated levels of individual PPAR-α activators. Then, MCP-1 and MKP-1 mRNA levels (A) and MCP-1 protein secretion into media (B) were determined using RT-PCR and ELISA, respectively. ELISA data are presented as means ± SEMs of three independent experiments (*p < 0.01 versus IFN-γ group). [file 1742-2094-9-34-S3.BMP]

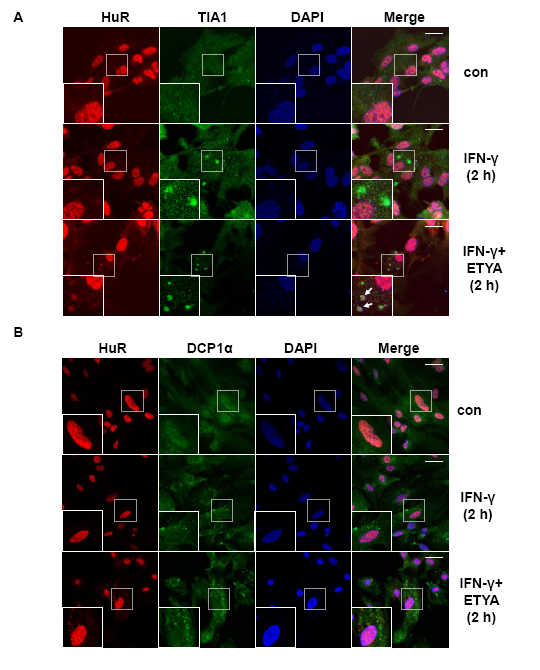

Supplement: Additional file 4 — Figure S4. ETYA promotes HuR translocation to stress granules (SGs), not processing bodies (PBs). (A and B) Confocal microscopic images of astrocytes immunostained with HuR, TIA1 (SG marker) (A), or DCP1α (PB marker) (B) under the indicated conditions. Insets are magnified images of the corresponding boxed regions. Scale bars, 20 μm. Arrows indicate co-localization of HuR and TIA1. [file 1742-2094-9-34-S4.BMP]

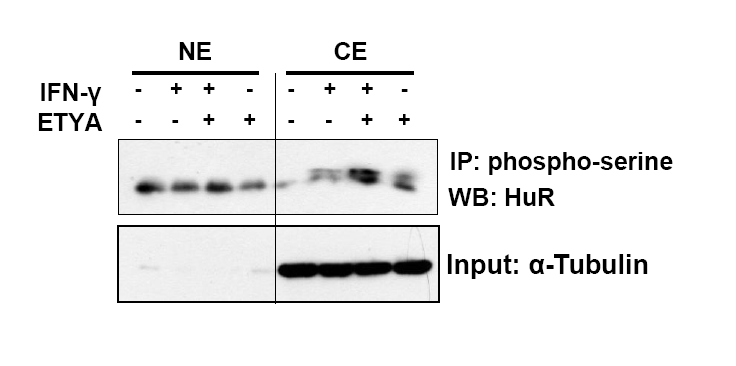

Supplement: Additional file 5 — Figure S5. ETYA increases HuR serine phosphorylation. Astrocytes were stimulated with IFN-γ in the presence or absence of ETYA, and nuclear extracts (NE) and cytosolic extracts (CE) were immunoprecipitated with an anti-phospho-serine antibody. Phosphorylation of HuR in immunoprecipitates was measured by Western blot analysis using an anti-HuR antibody. [file 1742-2094-9-34-S5.BMP]

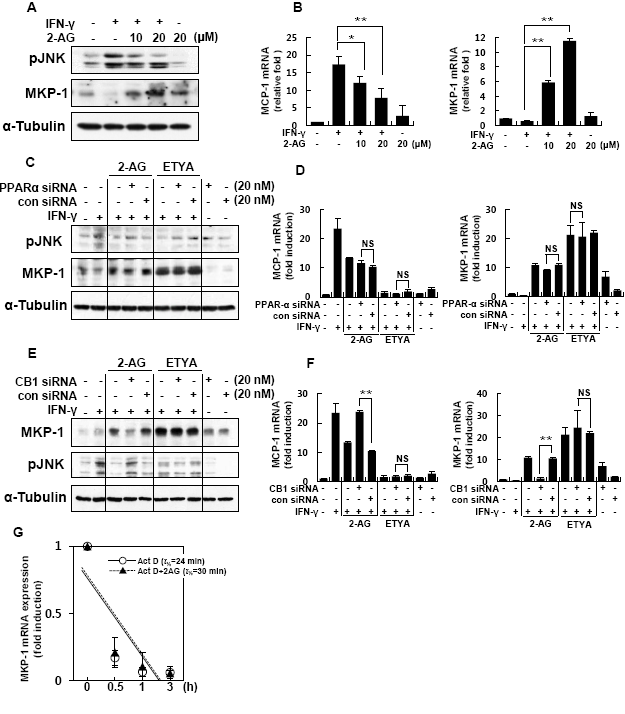

Supplement: Additional file 6 — Figure S6. 2-AG also suppresses CCL2/MCP-1 expression by inducing MKP-1 expression. (A and B) Astrocytes were stimulated with IFN-γ for 2 h in the absence or presence of the indicated dose of 2-AG. MKP-1 protein expression and JNK phosphorylation were analyzed by Western blotting (A), and MKP-1 and CCL2/MCP-1 transcript levels were examined by qRT-PCR (B). (C-F) Cells were transfected with siRNA duplexes specific for PPAR-α (C and D) or CB1 (E and F), and 48 h later were stimulated with IFN-γ for 2 h in the presence of 2-AG or ETYA. MKP-1 protein level and JNK phosphorylation were analyzed by Western blotting (C and E), and MKP-1 and CCL2/MCP-1 transcript levels were examined by qRT-PCR (D and F). (G) Astrocytes were treated with Act D for the indicated periods in the presence of 2-AG, after which MKP-1 mRNA levels were determined by qRT-PCR. [file 1742-2094-9-34-S6.BMP]
